# Impaired Cerebral Autoregulation Is Associated with Brain Atrophy and Worse Functional Status in Chronic Ischemic Stroke

**DOI:** 10.1371/journal.pone.0046794

**Published:** 2012-10-11

**Authors:** Mikio C. Aoi, Kun Hu, Men-Tzung Lo, Magdy Selim, Mette S. Olufsen, Vera Novak

**Affiliations:** 1 Biomathematics Program, Department of Mathematics, North Carolina State University, Raleigh, North Carolina, United States of America; 2 Division of Sleep Medicine, Brigham and Women's Hospital, Harvard Medical School, Boston, Massachusetts, United States of America; 3 The Research Center for Adaptive Data Analysis/Center for Dynamical Biomarker and Translational Medicine, National Central University, Chungli, Taiwan, Republic of China; 4 Department of Neurology, Beth Israel Deaconess Medical Center, Harvard Medical School, Boston, Massachusetts, United States of America; 5 Division of Gerontology, Beth Israel Deaconess Medical Center, Harvard Medical School, Boston, Massachusetts, United States of America; University of Queensland, Australia

## Abstract

Dynamic cerebral autoregulation (dCA) is impaired following stroke. However, the relationship between dCA, brain atrophy, and functional outcomes following stroke remains unclear. In this study, we aimed to determine whether impairment of dCA is associated with atrophy in specific regions or globally, thereby affecting daily functions in stroke patients.

We performed a retrospective analysis of 33 subjects with chronic infarctions in the middle cerebral artery territory, and 109 age-matched non-stroke subjects. dCA was assessed via the phase relationship between arterial blood pressure and cerebral blood flow velocity. Brain tissue volumes were quantified from MRI. Functional status was assessed by gait speed, instrumental activities of daily living (IADL), modified Rankin Scale, and NIH Stroke Score.

Compared to the non-stroke group, stroke subjects showed degraded dCA bilaterally, and showed gray matter atrophy in the frontal, parietal and temporal lobes ipsilateral to infarct. In stroke subjects, better dCA was associated with less temporal lobe gray matter atrophy on the infracted side (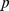 = 0.029), faster gait speed (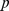 = 0.018) and lower IADL score (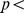
0.002). Our results indicate that better dynamic cerebral perfusion regulation is associated with less atrophy and better long-term functional status in older adults with chronic ischemic infarctions.

## Introduction

Cerebral autoregulation (CA) modulates cerebral blood flow in order to meet regional perfusion demands despite variations in arterial blood pressure (BP) associated with daily activities [1]. Dynamic CA (dCA) refers to the rapid response of cerebral vasculature to transient BP fluctuations. Several mechanisms are involved in dCA, regulating cerebrovascular resistance through dilation and constriction of cortical and pial arterioles [2]. Autoregulation is affected by age-related cerebro-microvascular diseases such as hypertension [3] and diabetes [4], and is damaged by ischemic stroke both acutely [5]–[8] and chronically [3].

Both impaired dCA [5], [9], assessed in the acute stroke period, and stroke-associated gray matter (GM) atrophy [10], [11] are associated with deficits in functional outcomes. However, the impact of chronically impaired dCA on brain atrophy, as well as its long-term effects on functional status in patients with ischemic stroke, remain unknown. If post-stroke dCA directly impacts GM atrophy and functional status, then interventions aimed at improving dCA function may provide an additional modality for clinicians to mitigate long-term functional deficits in stroke patients.

Noninvasive assessment of dCA often entails examining the coupling between continuous BP and cerebral blood flow velocity (BFV), measured by transcranial Doppler ultrasound (TCD). However, finding computational methods for the accurate quantification of this relationship is a challenge to reliable dCA assessment. Multimodal pressure-flow (MMPF) analysis [3], [12]–[15] can better quantify the nonlinear relationship between non-stationary BP and BFV signals than traditional transfer function methods [14] using spontaneous BP-BFV fluctuations during baseline conditions [13].

This study applied the MMPF-derived dCA measure to examine the relationship between dCA, regional brain tissue volumes, and functional status in a retrospective analysis of elderly subjects with chronic large vessel infarctions in the middle cerebral artery (MCA) territory, and in age-matched non-stroke subjects. We hypothesize that worse perfusion regulation is associated with enhanced gray matter atrophy in the temporal lobe, and worse long-term functional status in the elderly with chronic ischemic infarctions.

## Methods

### Experimental Protocals

#### Participants

All subjects signed informed consent and the study was approved by the Institutional Review Board at Beth Israel Deaconess Medical Center (BIDMC). Participants were recruited from community advertisement, Beth Israel Deaconess Medical Center, Joslin Diabetes Clinic patient registries and from the Harvard Cooperative Program on Aging research subject registry.

The data for this retrospective analysis of 142 subjects were selected from a database of records prospectively collected at the Syncope and Falls in the Elderly Laboratory and the Magnetic Resonance Imaging Center at BIDMC. The database was composed of records from three completed projects spanning January 2002 to February 2008: Cerebral vasoregulation in the elderly with stroke (March 2003–April 2005); Cerebral vasoregulation in diabetes (January 2002–December 2005); and Cerebral perfusion and cognitive decline in type 2 diabetes (January 2006–December 2008). Grant numbers and awarding institutions are provided in the financial disclosures section. All stroke subjects included in the current project were recruited for the vasoregulation in the elderly study while diabetic non-stroke subjects were from the vasoregulation in diabetes and cognitive decline in diabetes studies. Non-diabetic non-stroke subjects were recruited in all three studies. The subjects were selected for the present cohort, only if they completed both TCD and MRI measurements, and met the inclusion/exclusion criteria detailed below and in [4], [16].

Included stroke subjects had large vessel hemispheric MCA infarcts affecting 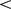
1/3 of the MCA territory documented by CT or MRI during the acute event, defined according to the Trial of ORG 10172 in Acute Stroke Treatment (TOAST) criteria [17] after completion of diagnostic work up, i.e., patients with MCA infarcts who had clinical and radiographic (duplex imaging or arteriography) findings of either significant (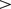
50%) stenosis or occlusion of an appropriate extracranial (i.e., carotid) or intracranial artery or branch cortical artery due to presumed atherosclerosis. Diagnostic studies excluded potential sources of cardioembolism. Clinical findings included those of cerebral cortical impairment (aphasia, neglect, or restricted motor involvement). A history of intermittent claudication, TIAs in the same vascular territory, a carotid bruit, or diminished pulses was used to support the clinical diagnosis. Infarcts greater than 1.5 cm in diameter on CT or MRI were considered to be of potential large-artery atherosclerotic origin. Subjects were required to be 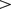
6 months post-stroke, in a clinically stable condition defined by a neurological exam, NIH Stroke Scale (NIHSS) 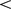
5, and able to walk unassisted (modified Rankin Scale (mRS)
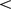
4) upon admission to the study.

All diabetic subjects were required to be diagnosed with type-II diabetes mellitus (DM) and to have been treated for at least 1 year prior to participation. Non-diabetic controls were age- and sex- matched to diabetic and stroke subjects from their respective studies with no clinical history of stroke and no focal deficits on neurological examination. Non-diabetic participants were required to have normal fasting glucose.

Subjects were excluded if they had intracranial or subarachnoid hemorrhage on MRI or CT or carotid artery stenosis (for control group, over 50% by medical history and MR angiobgraphy and for the stroke group, bilateral stenosis or stenosis contralateral to stroke). Other exclusion criteria included myocardial infarction within 6 months and other clinically important cardiac diseases; arrhythmias; significant nephropathy; kidney or liver transplant; renal or congestive heart failure; type I DM; or neurological or other systemic disorders. Incompatibility with 3 Tesla MRI, including claustrophobia, metal implants, pacemakers, and arterial stents was also an excluding factor.

Collectively, the three studies recruited 358 subjects (157 healthy controls, 115 diabetic controls, 86 stroke). We excluded 145 subjects (71 healthy control, 33 diabetic control, 41 stroke) because they either withdrew consent, they met exclusion criteria (listed above), or they did not get permission from their primary care provider. Of those subjects excluded, 24 subjects (14 healthy controls, 8 diabetic controls, 2 stroke) were excluded due to a poor temporal insonation window. From the remaining 213 subjects, 142 of them (52 healthy control, 57 diabetic control, 33 stroke) had complete TCD and MRI recordings and were used for the final analysis in the present study. Subjects with diabetes, hypertension, or both were included as part of the non-stroke group in order to control for dCA impairment associated with risk factors related to stroke. Demographic information of the selected cohort is listed in Table 1.

**Table 1:** Demographic and group summary statistics.

	Stroke	Control	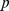 -value
Age (years)	63.4 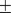 1.4	65.3 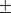 0.8	0.23
Male/Female	19/14	61/48	0.87
Race (W/A/AI/AA/U)	29/1/0/3/0	93/8/1/6/1	0.01*
Smokers (Y/N) (Current,Past)	(8/25), (28/5)	(4/72), (42/67)	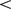 .01*, 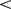 .01*
Packs per year	33.0 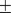 5.3	6.9 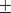 1.4	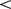 .01*
stroke side (R/L)(M,F)	(11/8),(7/7)	(38/23),(25/23)	0.74
Hypertens./Normotens.	23/10	36/73	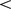 0.01*
Diabetes Mellitues (M/F)	0/0	35/22	–
Mean BP (mmHg)	87 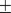 2	84 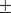 1	0.23
Systolic BP (mmHg)	131 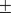 3	124 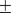 2	0.02*
Diastolic BP (mmHg)	63 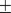 2	62 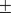 1	0.69
Body mass index (kg/m2)	27.7 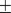 0.8	26.7 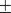 0.4	0.29
Gait Speed (m/s)	0.88 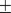 0.03	1.1 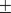 0.02	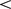 .01*
IADL (counts per score)	(6,10,4,7)	(33,21,3,2)	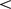 .01*
Mini mental state exam	26.7 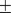 2.6	27.6 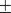 1.9	0.03*
NIH stroke scale	2.5 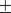 2.6	–	–
mRS (counts per 0,1,2,3,4)	(11,13,3,4,0)	–	–
BFV (NS/RND2) (cm/s)	39.4 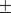 3.3	45.1 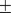 2.0	0.15
BFV (SS/RND1) (cm/s)	40.8 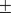 3.5	45.2 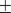 2.1	0.29
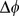 (NS/RND2) (degrees)	7.3 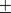 3.3	14.9 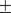 1.9	0.02*
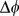 (SS/RND1) (degrees)	4.4 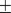 3.4	12.8 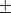 1.9	0.02*
CO_2_R (SS/RND1)	0.18 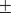 0.79	0.88 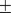 0.49	0.45
CO_2_R (NS)	0.94 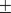 0.92	1.28 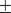 0.56	0.75
End-tidal CO_2_ (mmHg)	35.4 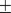 0.7	36.7 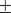 0.4	0.09
MCA (SS/RND1)(mm)	2.27 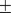 0.13	2.59 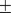 0.05	0.02*
MCA (NS/RND2)(mm)	2.44 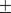 0.11	2.58 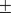 0.04	0.27
ICA (SS/RND1) (mm)	5.18 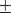 0.22	5.3 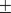 0.08	0.61
ICA (NS/RND2) (mm)	5.18 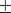 0.17	5.3 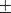 0.07	0.51
White blood cells (k/ul)	6.96 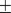 0.38	6.62 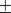 0.16	0.37
Hemoglobin (g/dL)	13.8 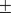 0.2	13.6 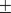 0.1	0.60
Hematocrit (%)	40.1 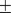 0.7	40.1 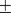 0.4	0.98
Cholesterol	179 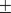 7	190 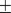 4	0.19
LDL (mg/dL)	95 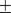 6	100 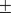 3	0.42
triglycerides (mg/dL)	147 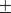 16	166 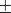 11	0.40
infarct (volume/ICV)*100	2.25 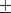 .43	–	–

Values are mean 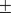 SE or number of subjects.

* indicates stroke and control groups are significantly different after controlling for false discovery rate. Abbreviations: W = white; A = Asian; AI = American Indian; AA = African American; U = unknown; R/L = right/left; M/F = male/female; IADL = instrumental activities of daily living; mRS = modified Rankin scale; BP = blood pressure; BFV = blood flow velocity; 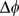 = BP-BFV phase difference; CO_2_R = CO_2_ reactivity; MCA = middle cerebral artery diameter; ICA = internal carotid artery diameter; ICV = intracranial volume; SS/RND1 = Stroke side; NS/RND2 = non-stroke side; LDL = Low density lipoprotein.

The stroke group consisted of 33 subjects that were 6.71
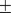
5.17 (mean 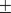 SE) years post acute event. The non-stroke group consisted of 109 age- and sex-matched individuals. There were 23 stroke and 36 non-stroke (12 control, 24 diabetic) participants with hypertension defined as receiving treatment for hypertension, or average BP
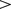
140 or 90 mmHg on 24-hour ambulatory home monitoring. There were 57 non-stroke participants receiving treatment for type 2 diabetes mellitus for 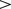
5 years. Antihypertensive medications were tapered and withdrawn for 3 days prior to the study. Glycemic control medications were allowed.

#### Transcranial Doppler Studies

Experiments were conducted in the morning after a thirty-minute rest during instrumentation. Baseline recordings of 5–10 minutes were collected during resting conditions when subjects were supine, awake and breathing regularly at their normal respiratory frequency. Vasoreactivity to CO_2_ (CO_2_R) was measured using 3 minutes of hyperventilation followed by 3 minutes re-breathing 5% CO_2_ in an air bag. Vasoreactivity was calculated as the slope of the regression of CO_2_ on BFV over baseline, hyperventilation, and rebreathing conditions. BFVs in both MCAs were measured from trans-temporal windows using TCD (MultiDop X4; Neuroscan, Sterling, VA). BP was recorded from the finger using the volume-clamp technique with a Finapres device (Finapres, Ohmeda Monitoring Systems, Englewood, CO) and corroborated by sphygmomanometer measurements. BP, BFV, respiration and end-tidal CO_2_ measurements (Capnomac Ultima, Ohmeda Monitoring Systems, Englewood, CO) were recorded at 500 HZ. Signals were decimated to 50 Hz before analysis.

#### Brain volumes and magnetic resonance imaging

The MRI studies were performed on a 3-Tesla GE Signa Vhi or Excite MRI scanner using a quadrature and phase array head coils (GE Medical Systems, Milwaukee, WI). Anatomical 3D magnetization prepared rapid gradient echo (MP-RAGE) images were used to quantify brain volumes with the Statistical Parametric Mapping software (SPM, University College,A London, UK) using spatial normalization and tissue classification. An anatomical template (Laboratory of Neuro Imaging, University of California, Los Angles, USA) was applied to measure GM and white matter (WM) in frontal, temporal, parietal, and occipital lobes. Normalized volumes (regional volume/global intracranial volume, cm^3^/cm^3^) of GM, and WM were used for analysis. Vessel diameters were derived from 3D MR angiography (time of flight, TOF) using the Medical Image Processing, Analysis, and Visualization (MIPAV) software from the Biomedical Imaging Research Services Section, NIH, Bethesda, MD, at 3 locations and averaged. Diameters Internal carotid arteries (ICA) and MCAs were computed from a single-slice transverse view with conservatively estimated accuracy (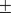
0.4 mm), based on the image resolution.

#### Functional Status

Functional status was assessed in both stroke and non-stroke groups by gait speed (measured by a 12-minute walking test at preferred walking speed), Instrumental Activities of Daily Living [18] (IADL) survey, and the Mini Mental State Exam (MMSE). The stroke group was also assessed by NIHSS, and mRS.

### Data Analysis

#### Cerebral autoregulation

Multimodal pressure-flow (MMPF) analysis was used for non-invasive assessment of dCA. Details on the development and performance of the method have been published previously [3], [12], [13].

The MMPF analysis for this study was performed according to the following four major steps:

1. Decomposition of BP and BFV signals into multiple empirical modes.Central to the MMPF method is the Hilbert-Huang transform [19]. This approach decomposes the original signal into empirical modes by a “sifting” algorithm [20] which adaptively extracts narrow-band, zero mean (but not necessarily stationary) components of the original BP and BFV time series.
2. Selection of empirical modes for dominant oscillations in BP at 0.1–0.3 Hz and corresponding oscillations in BFV.In order to determine a meaningful phase relationship between the BP and BFV time series, empirical modes from both series must be selected from within the same frequency band. Previous studies have shown that dCA can be assessed from respiratory-induced pressure-flow variations during spontaneous respiration [12], [13], [21], [22]. Therefore, the modes used for further analysis were selected in order to correspond to the respiratory frequency range of 0.1–0.3 Hz.
3. Calculation of instantaneous phases of extracted BP and BFV oscillations.Since each empirical mode has zero mean and is sufficiently narrow-band, the complex part of each mode can be calculated by the Hilbert transform [19]. The instantaneous phase of BP or BFV oscillations is obtained from the inverse tangent transform of the ratio of the real and complex parts of the signal.
4. Calculation of the mean BP-BFV phase difference (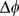), as the dCA measure.The arithmetic mean of the difference in the phases of extracted BP and BFV oscillations was calculated and served as the metric by which we assessed dCA. Larger 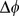 corresponds to better dCA function [3], [21], [22].

#### Statistical Analysis

For all subjects, MRI and TCD measurements were made on both left and right sides and analyses were conducted by stroke side and non-stroke side. Since non-stroke subjects did not have an affected side, each non-stroke subject was randomly assigned a ÒstrokeÓ side (RND1) and a Ònon-strokeÓ side (RND2). The side assignment was implemented in order to have a left-side-stroke/right-side-stroke probability that approximately matched the distribution of the stroke group. Univariate group differences were determined by one -, or two-tailed t-test, or 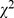 test where appropriate. Within-group differences between sides in brain volumes, blood flow velocity, the dCA measure (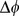), and ICA and MCA diameters were determined by 1-sided Wilcoxon signed-rank test.

To constrain the number of variables under consideration, regression analyses were limited to those areas directly affected by stroke (i.e. MCA territory on the stroke side/RND1). Linear regression models were tested for the effects of 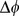 on GM volumes, 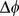 on functional measures and GM volumes on functional measures. With each model, we examined the primary independent variable (either 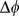 or GM volume) was examined for a significant effect separately for stroke and non-stroke subjects.

Regression parameters were estimated using the traditional least squares estimator as well as the Theil-Sen robust regression estimator [23], [24] with outlier skipping by the orthogonal projection method (TSOP). This robust regression estimator down-weights response outliers and high leverage points observed in our data [25].

Robust regression parameter inference, including simultaneous 95% confidence intervals and hypothesis test statistics, was estimated by bootstrapping of observations. For each robust regression, bootstrapping consisted of n = 600 resamples of the stroke group, with replacement, of the multivariate observations. Regression parameter estimates, standard errors, bootstrap confidence intervals, and hypothesis test statistics were calculated using functions written by R.R. Wilcox [25] in the rallfun.v-14 package (downloaded from www-rcf.usc.edu/rwilcox/ on 6/19/11) for the statistical computing software R [26]. The Theil-Sen estimates were calculated using the tsreg() function embedded within the opreg() function. Bootstrap confidence intervals were calculated using the regci() function. Default settings were used for all functions. Linear models for robust regression controlled for age, mean blood pressure, sex, BMI and infarct volume.

Relationships between GM and IADL and between 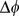 and IADL were tested by logistic regression. The IADL scores 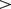
3, which were present in 7 subjects, were grouped into a single category. Statistical control variables were included based on the presence of significant correlations with brain matter volumes, functional status measurements or 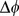. The resulting models were adjusted for age, sex, mean BP, infarct volume, and body mass index (BMI).

For groups of hypothesis tests that included multiple variables (differences between groups and between sides), the threshold of significance was adjusted to maintain a false discovery rate [27] of 0.05. Statistical inference was computed in JMP (SAS Institute. Cary, NC, USA).

## Results

### Effects of stroke on functional status, brain volumes, and dCA

Demographic characteristics, mean BP, MCA and ICA diameters on the non-stroke side, mean BFV and CO_2_ vasoreactivity and laboratory results were similar between the stroke and non-stroke groups (Table 1). Stroke subjects had worse scores on performance and cognitive measures (IADL 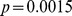, MMSE 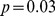) and slower gait speed (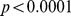). In stroke subjects, frontal, temporal and parietal lobe volumes were significantly smaller on the stroke side as compared to the non-stroke side, and to the RND1 and RND2 sides of non-stroke subjects (Table 2). Mean BFV and CO_2_ vasoreactivity was not significantly different between sides within either group. There were no significant differences in any measures between the RND1 and RND2 sides of non-stroke subjects.

**Table 2:** Brain volumes by region and side.

	Stroke Group		Control Group	
Region	S side	NS side	RND1	RND2
Front. GM	5.2 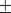 0.10	5.6 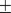 0.09*	5.5 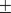 0.05*	5.5 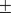 0.05
Temp. GM	4.4 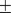 0.07	4.7 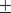 0.06*	4.6 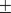 0.04*	4.7 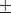 0.03
Par. GM	3.0 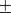 0.06	3.2 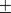 0.05*	3.2 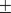 0.03*	3.2 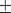 0.03
Occ. GM	1.8 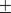 0.03	1.8 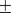 0.03	1.8 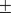 0.02	1.8 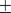 0.02
Temp. WM	2.0 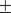 0.04	2.1 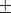 0.05*	2.1 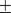 0.02*	2.1 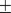 0.03
Front.WM	4.1 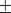 0.10	4.5 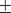 0.09*	4.4 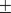 0.06*	4.4 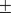 0.05
Par. WM	2.5 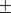 0.06	2.6 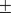 0.06*	2.7 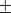 0.03*	2.7 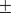 0.03
Occ. WM	1.7 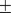 0.03	1.1 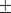 3.13*	1.8 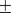 0.03	1.8 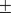 0.03

Gray matter (GM) and white matter (WM) volumes for stroke (S) and non-stroke (NS) groups by side. Values are mean
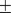
SE.

* indicates values are significantly different from stroke side of stroke group by Wilcoxon signed-rank test, with false discovery rate of 0.05.

BP-BFV phase shift (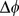) was smaller in stroke subjects compared to non-stroke subjects (Table 1, stroke side: 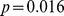; non-stroke side: 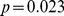), but was not different between stroke and non-stroke sides. In the non-stroke group, non-diabetic subjects had larger 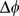 than diabetic subjects (RND1: 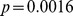; RND2: 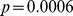), who had similar 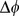 to stroke subjects. BP-BFV phase shift was not significantly associated with age; mean, systolic, or diastolic BP; hypertension diagnoses or treatment; mean BFV; CO_2_ vasoreactivity; left/right stroke side; glucose levels; hematocrit; hemoglobin A1C levels; BMI; ICA or MCA diameters; total cholesterol, LDL or triglycerides.

The fraction of subjects with either current or previous smoking history was significantly different for stroke and non stroke groups (Table 1). Neither smoking status nor packs per year (pack years) were significantly correlated (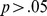) with gait speed, 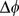 or GM volumes. Therefore, smoking data was not included in further analyses.

### Associations between dCA, regional brain volumes, and functional status

The main results for least squares regressions are summarized in Table 3, and for robust regressions in Table 4.

**Table 3:** Least-squares results for model fit, and effect tests for 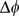.

Response	Predictor	Group	Model fit	Effect test
TGM	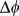	Stroke	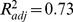 , 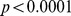	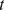 -ratio 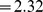 , 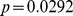
		Non-stroke	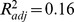 , 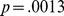	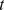 -ratio 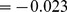 , 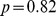
Gait	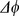	Stroke	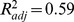 , 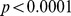	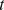 -ratio 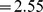 , 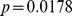
		Non-stroke	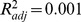 , 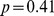	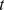 -ratio 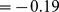 , 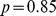
IADL	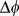	Stroke	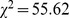 , 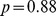	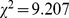 , 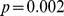
		Non-stroke	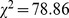 , 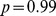	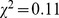 , 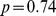

Regression on temporal lobe grey matter (TGM), gait speed (Gait), instrumental activities of daily living survey (IADL). Estimates were calculated with regression equations controlling for age, BMI, mean arterial pressure, sex, and infarct volume. The 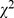 tests for IADL correspond to lack of fit, and likelihood ratio tests.

**Table 4:** Robust regression bootstrap estimates.

Response	Predictor	Estimate	S.E.	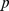 -value
TGM	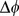	6.8 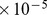	3.97 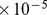	0.047
Gait	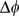	4.05 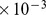	3.04 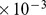	0.097
Gait	TGM	30.0	23.0	0.080
Gait	TGM*	28.9	14.7	0.060

Theil-Sen estimates for regressions with 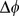, temporal lobe gray matter volume (TGM), and gait speed (Gait) for stroke subjects. Estimates were calculated with regression equations controlling for age, BMI, mean arterial pressure, sex, and infarct volume.

* indicates results without controlling for infarct volume.

#### Pressure-flow phase shift and brain volume

**Figure 1:**
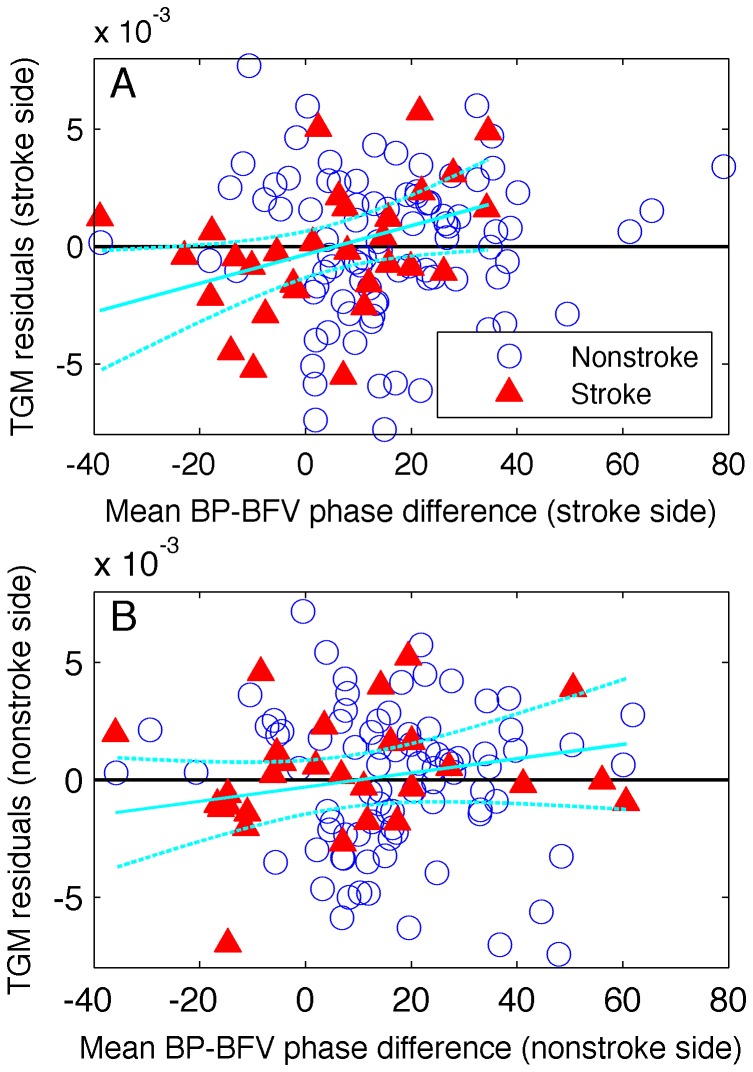
Effect of 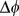 on temporal lobe gray matter volume. Residuals of least squares regression on relative temporal lobe gray matter (GM) volume against BP-BFV phase difference (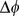). Regression included age, BMI, mean BP, sex and infarct volume for stroke side of the stroke group plotted against blood pressure-blood flow velocity (BP-BFV) phase difference for stroke (**A**) and non-stroke (**B**) sides. Cyan lines indicate least squares regression line for BP-BFV phase difference on temporal GM residuals in stroke subjects, and 95% prediction interval.

Using least squares regression, a larger 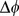 was associated with larger temporal lobe GM volume in stroke subjects (model fit: adjusted 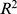 (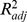) = 0.73, 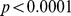, effect test: 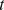-ratio 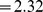, 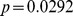) after controlling for age, sex, mean BP, BMI, and infarct volume, but not in non-stroke subjects (model fit: 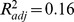, 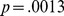, Effect test: 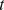-ratio 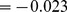, 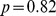). The relationship between 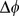 and temporal lobe GM, independent of age, sex, mean BP, BMI, and infarct volume is shown in Fig. 1. There were no significant associations between 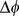 and temporal WM or between 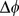 and GM or WM volumes in frontal, parietal, or occipital regions.

In agreement with the least squares estimates, the Theil-Sen estimates showed a significant effect (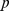 = 0.047) of 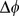 on temporal lobe GM for stroke subjects (Table 4) after controlling for age, sex, mean BP, BMI, and infarct volume, but not for other brain regions.

#### Pressure-flow phase shift and functional status

**Figure 2:**
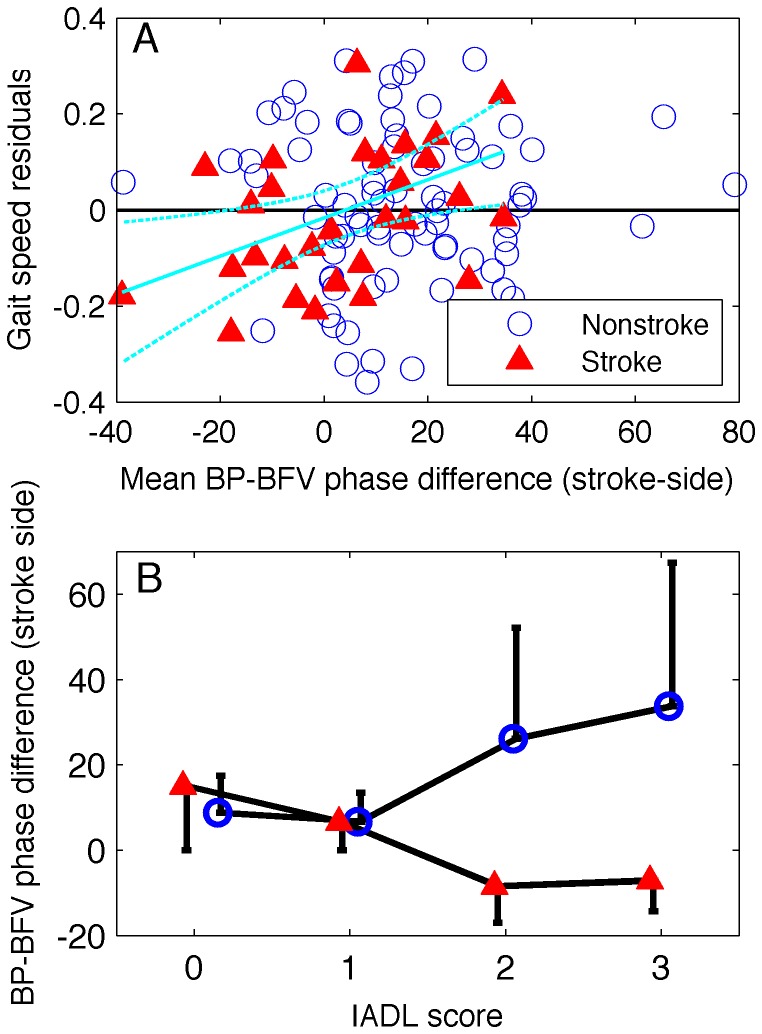
Effect of 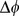 on functional status. **A**: the residuals from least squares regression of age, BMI, mean BP, sex and infarct volume for stroke side of the stroke group, on gait speed plotted against stroke-side blood pressure-blood flow velocity (BP-BFV) phase difference. Cyan lines indicates least squares regression for BP-BFV phase difference on gait speed residuals in stroke subjects and 95% prediction interval. **B**: BP-BFV phase difference for stroke and non-stroke subjects as a function of score on the instrumental activities of daily living (IADL) survey. Error bars indicate standard error.

Using the least squares estimator, a larger 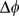 was associated with faster gait speed for stroke subjects (model fit: 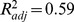, 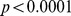, Effect test: 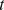-ratio 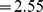, 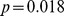) after controlling for age, sex, mean BP, BMI, and infarct volume, but not for non-stroke subjects (model fit: 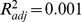, 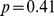, Effect test: 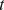-ratio 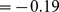, 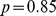). The relationship between 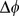 and gait speed, independent of age, sex, mean BP, BMI, and infarct volume is shown in Fig. 2A.

The Theil-Sen estimate for the relationship between 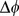 and gait speed did not meet the threshold for a significant effect, but was found to be marginally significant (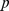 = 0.097, Table 4) when controlling for age, sex, mean BP, BMI and infarct volume. The 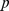-value of the test was considerably reduced (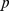 = 0.067) when infarct volume was not included in the regression.

Ordinal logistic regression showed that a larger 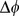 was associated with a better daily functionality (i.e., lower IADL) for stroke subjects (Lack of fit: 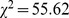, 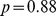, Effect likelihood ratio test: 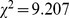, 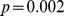) (Fig. 2B) after controlling for age, sex, mean BP, BMI and infarct volume but there was no significant association between 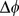 and IADL in non-stroke subjects (Lack of fit: 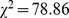, 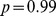, Effect likelihood ratio test: 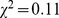, 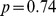). No associations were found between 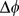 and MMSE, mRS or NIHSS.

#### Regional brain volumes and functional status

In contrast with our findings of associations between 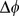 and temporal GM, and between 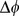 and functional status in stroke subjects, no significant associations were found between temporal GM and gait speed (model fit: 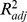 = 0.51, 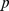 = 0.0004, Effect test: 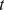-ratio 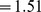, 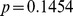) after controlling for age, sex, mean BP, BMI and infarct volume using the least squares estimator. However, faster gait speed was associated with larger temporal lobe GM volumes if infarct volume was not included in the regression equation for both stroke (model fit: 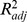 = 0.62, 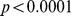, Effect test: 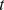-ratio 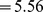, 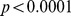) and non-stroke (model fit: 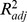 = 0.12, 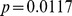, Effect test: 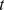-ratio 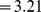, 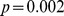) subjects. This effect was significantly stronger in stroke subjects than for non-stroke subjects (Effect test of interaction: 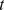-ratio 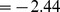, 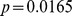).

Similar to our results with least squares regression, the Theil-Sen estimate for a relationship between gait speed and temporal GM was marginally significant, (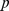 = 0.08) when infarct volume was included, with a smaller 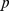-value (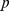 = 0.06) when infarct volume was not included.

IADL was not significantly associated with temporal GM (Lack of fit: 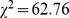, 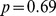, Effect likelihood ratio test: 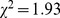, 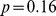) after controlling for age, sex, mean BP, BMI and infarct volume. However, larger temporal lobe GM on the stroke side was associated with better functionality (i.e. lower IADL) for stroke subjects (Lack of fit: 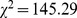, 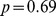, Effect likelihood ratio test: 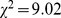, 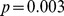) but not for non-stroke subjects (Lack of fit: 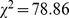, 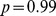, Effect likelihood ratio test: 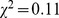, 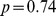). Brain volumes were not associated with MMSE or NIHSS.

#### Regional brain volumes, gait speed, and infarct volume

In order to examine the confounding effect of infarct volume on linear regressions observed above, linear regressions of infarct volume on gait speed and temporal lobe GM were examined, after correcting for age, sex, mean BP and BMI. Least squares regression showed that larger infarct volumes were associated with significantly slower gait speed (model fit: 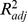 = 0.505, 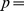
0.0004, Effect test: 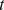-ratio = −5.31, 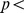
0.0001) and smaller temporal GM volumes (model fit: 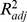 = 0.68, 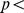
0.0001, Effect test: 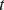-ratio = −7.93, 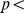
0.0001). On the other hand, robust regression showed only a modest effect of infarct volume on temporal GM (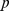 = 0.077) and none for gait speed (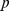 = 0.51).

## Discussion

This study examined the relationships among dCA, brain structural volumes, and functional status in subjects with chronic ischemic stroke using a nonlinear dCA assessment computed using the MMPF method. Both traditional least squares regression and robust regression were used to test the hypothesis that better dCA function is associated with less GM atrophy and better functional status. Supporting this hypothesis, smaller 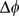 was associated with smaller temporal lobe GM, slower gait speeds, and higher IADL (lower function), independent of age, sex, mean BP, and BMI.

### Clinical implications

Poor clinical outcomes have been shown to be associated with impaired dCA following both brain injury [28]–[30] and acute ischemic stroke [5], [9]. This study presented evidence indicating that not only is worse functional status post-stroke concomitant with impaired dCA, but the degree of dCA impairment is negatively correlated with functional performance (Fig. 2).

Atrophy of brain tissue continues following the acute stroke period and extends from periinfarct zones to contralateral and remote cortical and subcortical regions that are functionally connected to the infarct site [31]. Supporting this notion are findings of impaired vascular reactivity in regions distant from the infarct site in patients with chronic ischemic infarctions [16]. We also noted a consistent pattern of GM atrophy, independent of the specific infarct location, in the ipsilateral frontal, parietal and temporal lobes extending beyond the affected MCA territory (Table 2).

Both impaired CA [9] and GM atrophy [10], [11] following stroke have been associated with cognitive impairment. Therefore, chronic impairment of autoregulation may affect perfusion redistribution during daily activities [4], [32]–[34], contributing to GM atrophy and influencing long-term recovery after stroke.

In the present study, although all MCA-territory brain regions showed significant atrophy for stroke subjects, the relationships between dCA impairment, GM atrophy, and functional status were most prominent for temporal lobe GM. These relationships were not observed in age-matched, diabetic, or non-diabetic non-stroke subjects, and were independent of BP. However, diabetic subjects also had impaired dCA compared to healthy nonstroke subjects, which suggests that associations between dCA, functional impairment, and temporal lobe GM are stroke-specific and not due to normal aging or stroke-independent dCA impairment.

Temporal lobe structures such as the insular cortex and amygdala are key centers of the autonomic network [35], playing a role in vascular resistance and sympathetic modulation of dCA [36]. Both autoregulation [3], [6]–[8] and autonomic regulation [37], [38] are altered following stroke, and sympathetic activity has been shown to affect dCA [39], although its precise role is contended [40], [41]. Damage to the insular cortex in particular is associated with autonomic dysfunction [35], [36], [42] and poor long-term prognosis [42] in stroke subjects. Medial temporal lobe atrophy is also associated with greater incidence of post-stroke dementia [11]. Thus, temporal lobe atrophy may persistently affect dCA via its influences on autonomic function, in addition to its more direct effects on functional performance. A more detailed analysis of the temporal lobe structures is needed to determine if specific regions of the temporal lobe are responsible for its role in the dCA -temporal GM-functional status relationship found in the current study.

Disrupted autonomic regulation after stroke may also influence the clinical outcomes measured in this study, independent from dCA. Sympathetic reflex activity is attenuated with chronic stroke and this attenuation is correlated with functional motor capacity [43]. Similarly, pathological sympathetic activity has been shown to be associated with poor long-term outcomes in subjects with thromboembolic stroke [42]. The potential role of autonomic function on the dCA - brain atrophy - functional outcomes relationship should be directly examined in stroke subjects. In this regard, future studies should consider BP-BFV coupling over frequency bands more directly associated with autonomic activity [44].

We hypothesize that treatments aimed at the improvement of dCA may play a role in optimizing the functional performance and quality of life in elderly people with chronic ischemic stroke. Therefore, the dCA status and the potential effects of treatments (such as antihypertensive medications [45], [46]) on dCA function and functional outcomes should be considered in the overall treatment strategy for patients with chronic stroke. For example, angiotensin receptor blockers (ARB's) have been shown to prevent vascular dementia in elderly subjects [47] and brain atrophy in hypertensive subjects [48], and they are significantly more effective at preventing stroke in hypertensive patients than other blood-pressure-lowering medications [49]. They have not been shown to lower risk of recurrent stroke, however, [50]. Since some component of the neuroprotective effects of ARBs may be independent of their BP-lowering effects [51], [52], the neuroprotective effects of ARBs may be acting through their ability to improve autoregulatory function. Therefore if, as the present study suggests, there is a connection between dCA, brain atrophy and functional status following stroke, treatment with ARBs may be an effective strategy for stroke patients to preserve functional status, even in the absence of hypertension. While ARB's have been shown to improve static cerebral autoregulation in hypertensive rats [53], investigations of the effects of ARBs on dCA are needed.

### Limitations

The results of least squares regression were in agreement with robust regression estimates for the relationship between 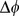 and temporal lobe GM. However, in part due to the presence of outliers, the associations between 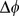 and gait speed were more tenuous, and the correlation between temporal lobe GM and gait speed was only marginally significant based on robust regression. The latter results are surprising, as degree of GM atrophy is known to coincide with degree of functional impairment. For instance, Lee 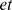 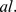 [54] demonstrated that brain atrophy negatively impacts functional recovery, as measured by mRS, where “acceptable” outcomes were operationalized as mRS 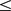
4. In the current study, mRS 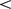
4 was a necessary condition of inclusion. The Lee 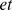 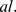 sample also included individuals with more severe strokes than in the current study, as measured by NIHSS. Furthermore, the number of participants with IADL scores larger than 2 was limited in our study. Therefore, a larger prospective study that draws upon subjects from a population-based sample of people with various types of strokes may be beneficial to verifying the findings of this study.

Including infarct volume in the linear models had a considerable effect on robust regression results. When including infarct volume, the regressions of 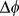 on gait speed and temporal lobe GM on gait speed were marginally significant (0.05
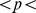
0.1) These associations were somewhat stronger when infarct volume was excluded from the models (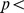
0.07 for both). Robust regression estimators tend to have poor asymptotic efficiency [25]. Consequently, the small sample size of this study makes our results susceptible to type II error, particularly with a larger number of covariates. Thus, the efficiency of the estimator may help explain the impact of infarct volume on the models in spite of the lack of a robust association between gait speed and infarct volume (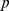 = 0.51). Therefore, we view our results with both the least squares and robust regression estimators to be in support of our hypothesis that there is an interaction between dCA, brain atrophy, and functional status following stroke, although we concede that studies with larger and more diverse samples of stroke subjects are necessary in order to verify our results.

The size and variability of the sample may also explain why no significant differences in CO_2_ reactivity between groups were detected (Table 1). In agreement with previous findings of depressed CO_2_ reactivity for stroke subjects [55], the group mean of CO_2_ reactivity was lower in stroke subjects than in non-stroke subjects. However, the between-group difference was not significant due to the large standard errors within both groups.

Due to the retrospective nature of the present study, the sample selected for analysis was not population-based. Specifically, our non-stroke group included individuals with diabetes, hypertension, or both while all stroke subjects were non-diabetic. However, diabetes is a major risk factor for stroke and hypertension [56], [57]. Cardiovascular risk factors, such as hypertension and diabetes are known to alter endothelial function and have adverse effects on cerebral vasoreactivity. Both diabetes and hypertension are shown to degrade dCA [3], [22], and dCA was impaired in diabetic subjects in the present study. Importantly, recent work has suggested that impairment of CA precedes stroke [58], [59]. Inclusion of individuals with risk factors for stroke (e.g., DM and hypertension) within the control group, but not in the stroke group, allowed us to determine the extent to which our results were a reflection of the interactions between dCA impairment and effects due to stroke versus the interactions between dCA impairment, stroke and effects due to cardiovascular risk factors that are predictive of stroke. Thus, it is conceivable that preexisting impairment of CA may influence GM atrophy and functional recovery following stroke. Leveraging the notion that dCA impairment precedes stroke, we may posit that impairment of dCA may alter neurovascular coupling in GM, thus making the stroke-affected regions vulnerable to transient cerebral blood flow fluctuations, and altering processes governing connectivity for neuroregeneration and functional recovery. Future work should include the analysis of a population-based sample of larger size.

The study design has also limited the elucidation of causality between impaired cerebral autoregulation, brain tissue loss and poor functional outcomes in stroke patients. Since stroke itself can cause impairment of dCA, brain tissue loss and worse functional outcomes, the cross-sectional study design cannot demonstrate a causal link between dCA, tissue loss and functional outcome. Therefore, future work should include a longitudinal study that would evaluate the time course and relationship between cerebral autoregulation and functional outcomes.

## Conclusion

There are likely associations between dCA, temporal lobe GM, gait speed, and IADL, indicating that dCA may impact GM atrophy and functional recovery following stroke. The relationships between dCA, temporal lobe GM and functional status were independent of age, sex, BMI, mean BP and mean BFV, but it is unclear how infarct volume is associated with these parameters. Therefore, dCA impairment may be an important factor underlying perfusion adaptation to daily activities and progression of regional atrophy and functional recovery in patients with stroke.
